# MicroRNA‐203 mimics age‐related aortic smooth muscle dysfunction of cytoskeletal pathways

**DOI:** 10.1111/jcmm.12940

**Published:** 2016-08-09

**Authors:** Christopher J. Nicholson, Francesca Seta, Sophie Lee, Kathleen G. Morgan

**Affiliations:** ^1^ Department of Health SciencesBoston UniversityBostonMAUSA; ^2^ Department of MedicineBoston University School of MedicineBostonMAUSA

**Keywords:** focal adhesion, aortic stiffness, microRNA, vascular smooth muscle, cytoskeleton

## Abstract

Increased aortic stiffness is a biomarker for subsequent adverse cardiovascular events. We have previously reported that vascular smooth muscle Src‐dependent cytoskeletal remodelling, which contributes to aortic plasticity, is impaired with ageing. Here, we use a multi‐scale approach to determine the molecular mechanisms behind defective Src‐dependent signalling in an aged C57BL/6 male mouse model. Increased aortic stiffness, as measured *in vivo* by pulse wave velocity, was found to have a comparable time course to that in humans. Bioinformatic analyses predicted several miRs to regulate Src‐dependent cytoskeletal remodelling. qRT‐PCR was used to determine the relative levels of predicted miRs in aortas and, notably, the expression of miR‐203 increased almost twofold in aged aorta. Increased miR‐203 expression was associated with a decrease in both mRNA and protein expression of Src, caveolin‐1 and paxillin in aged aorta. Probing with phospho‐specific antibodies confirmed that overexpression of miR‐203 significantly attenuated Src and extracellular signal regulated kinase (ERK) signalling, which we have previously found to regulate vascular smooth muscle stiffness. In addition, transfection of miR‐203 into aortic tissue from young mice increased phenylephrine‐induced aortic stiffness *ex vivo*, mimicking the aged phenotype. Upstream of miR‐203, we found that DNA methyltransferases (DNMT) 1, 3a, and 3b are also significantly decreased in the aged mouse aorta and that DNMT inhibition significantly increases miR‐203 expression. Thus, the age‐induced increase in miR‐203 may be caused by epigenetic promoter hypomethylation in the aorta. These findings indicate that miR‐203 promotes a re‐programming of Src/ERK signalling pathways in vascular smooth muscle, impairing the regulation of stiffness in aged aorta.

## Introduction

Cardiovascular diseases are the leading cause of death worldwide 1. In the vascular system, the proximal aorta plays a critical role as a shock absorber against the intermittent pulsatile output from the heart, and provides a steady flow to smaller vessels downstream 2, 3. With age, the proximal aorta stiffens and this shock absorption function is lost, sending higher pressures to small downstream vessels in the kidney, brain and heart, which predicts for hypertension and end organ damage 4, 5, 6, 7, 8.

Vascular smooth muscle cell (VSMC) contraction is predominantly initiated by Ca^2+^‐dependent activation of myosin light chain kinase, which phosphorylates the myosin light chains, leading to formation of actomyosin cross‐bridges and increased VSMC stiffness 9. It is now well‐established, however, that the actin cytoskeleton 10, 11, 12, 13, 14, 15 and focal adhesion (FA) complexes 2, 15, 16, 17, 18, 19 influence the output of the contractile machinery to regulate VSMC stiffness. This is achieved through the connection between the FAs and the actin cytoskeleton, which, through the integrins, allow force to be transmitted to the extracellular matrix (ECM) and the tissue as a whole 20, 21. In recent years, we and others have determined a role for FA/actin signalling and remodelling in the regulation of VSMC stiffness, which has emerged as an important contributor to aortic stiffness 2, 22, 23, 24, 25, 26, 27. This process is regulated through activation of the non‐receptor tyrosine kinase Src, which promotes tyrosine phosphorylation of focal adhesion kinase (FAK), paxillin and crk‐associated substrate (CAS), leading to FA remodelling and recycling through an endocytic pathway 17, 24, 28. Furthermore, FA proteins act as extracellular signal regulated kinase (ERK) scaffolds, mediating ERK activation, which was recently shown to be involved in VSMC stiffness development 24. Importantly, previous studies from our laboratory demonstrated that Src‐dependent tyrosine phosphorylation of FA proteins is essentially abolished in aged mouse aorta 2. Moreover, although Src inhibition dramatically decreases agonist‐induced VSMC contractility and stiffness in young mice, it has no such effect in the aorta from aged mice. These results suggest that this Src‐dependent FA pathway is lost in aged aorta, interfering with the dynamic regulation of stiffness 2. The molecular basis behind this age‐related impairment of FA signalling in vascular smooth muscle has not been determined.

Recently, microRNAs (miRs) have emerged as important regulators of VSMC development and function 29, 30, 31, 32, 33, 34, 35, 36, 37, 38. MicroRNAs are a family of short (21–25 nucleotide) RNAs, which typically negatively regulate protein translation of mRNA targets by repressing translation and/or promoting mRNA degradation 39. Mammalian miRs interact with the 3′‐untranslated region (3′‐UTR) of mRNA through a short (6‐8 nucleotide) ‘seed’ sequence, and may therefore regulate the expression of hundreds of targets 39 and drive an entire program of phenotypic function. Critically, miRs have been reported to be dysregulated in cardiovascular diseases, such as hypertension, atherosclerosis and aortic aneurysms 30, 40.

In the current study, we sought to determine whether aberrant expression of miRs could play a role in the age‐related impairment of Src‐mediated plasticity of aortic smooth muscle. We tested the hypothesis that miRs regulate the expression of members of a Src signalling program in contractile vascular smooth muscle, contributing to the aged phenotype. We report here that age‐related upregulation of miR‐203, in a cause‐and‐effect manner, impairs key phosphorylation events downstream of Src tyrosine kinase, which is associated with increased tissue stiffness. We conclude that changes in miR‐203 expression play a pivotal role in defective regulation of stiffness in aged mouse aorta.

## Materials and methods

### Ethical approval

All procedures were performed in accordance to protocols approved by the Institutional Animal Care and Use Committee of Boston University (Permit Number: A3316‐01). The animals were maintained according to the guidelines set out by the NIH Guide for the Care and Use of Laboratory Animals, and were obtained and used in compliance with federal, state, and local laws. Mice were killed by cervical dislocation following anaesthetization by isoflurane inhalation. For the pulse wave velocity (PWV) measurements, mice were deeply anaesthetized using isoflurane and maintained horizontally on a heated pad (38°C).

### *In vivo* pulse wave velocity measurements

**Figure 1:**
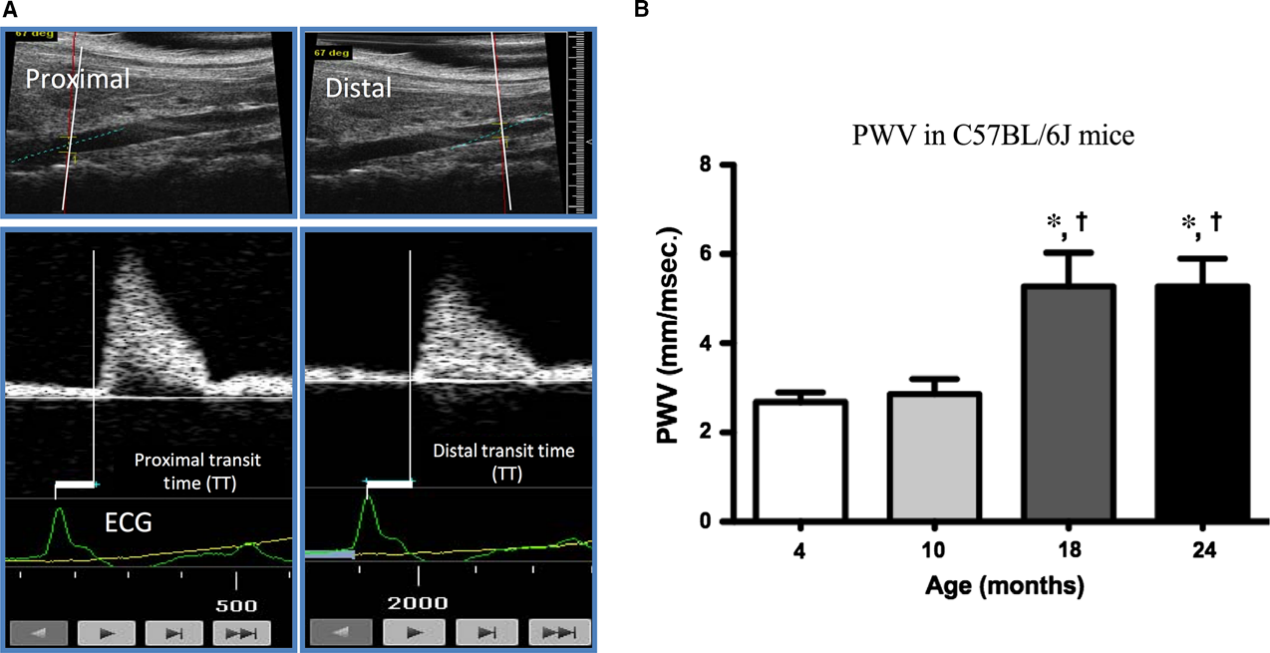
Aortic stiffness is increased in aged mice. Aortic stiffness assessed *in vivo* by pulse wave velocity (PWV). (**A**) Blood flow waveforms were continuously recorded for 20 sec. at a proximal and a distal location along the aorta with simultaneous ECG. PWV was calculated by dividing the distance between the proximal and distal locations by the difference between the proximal and distal transit times and expressed in mm/msec. (**B**) PWV was measured in 4‐ (*n* = 11), 10‐ (*n* = 9), 18‐ (*n* = 8) and 24‐month‐old (*n* = 7) male mice to obtain a time course of aortic stiffness development. *P* < 0.05 compared to 4 months (*) and 10 months (†) (two‐tailed Student's *t*‐test).

Pulse wave velocity, the standard *in vivo* measure of arterial stiffness, was performed as previously established 41 in 4‐, 10‐, 18‐ and 24‐month‐old male C57BL/6J mice. Briefly, PWV was measured by acquiring flow pressure waveforms from 2 locations along the aorta, one proximal, (at the level of the renal vein crossing over the aorta) and one approximately 1 cm distal, using high‐resolution Doppler echocardiography (VEVO770; FujiFilm, VisualSonics, Ontario, Canada). This procedure has been validated to record similar PWV measurements from the proximal aortic arch downwards 41. Waveforms were automatically documented for 20‐sec. of continuous recordings with simultaneous electrocardiogram (ECG), allowing assessment of the foot‐to‐foot transit times (TT; Fig. 1A). Pulse wave velocity was calculated by dividing the distance between the proximal and distal locations (in mm) by the difference in the proximal and distal TT of the waveforms (in msec.).

### Bioinformatic prediction tools

We employed a bioinformatics methodology to determine miRs of interest, using two miR prediction tools, Target Scan (http://www.targetscan.org) and miRbase (http://www.mirbase.org). These tools search for matches between the seed sequence of miRs and the 3′‐UTRs of mRNA targets.

### Preparation of aortic samples

Following euthanasia, aortas were quickly excised from young (3–4 months) and aged (24–29 months) mice and placed directly in ice‐cold tissue collection buffer (TCB; modified Krebs solution ‐ in mM: 154 NaCl, 5.4 KCl, 1.2 MgSO_4_, 10 MOPS, 5.5 glucose, and 1.6 CaCl_2_; pH = 7.4) 42. Vessels were cleaned from excess perivascular fat and 4–5 mm axial length rings were isolated from the proximal end of the thoracic aorta in preparation for stiffness measurements (see below). For biochemical analyses, tissues were quick‐frozen in an acetone‐dry ice slurry containing 10 mM dithiothreitol (for RNA‐based studies) or 10 mM dithiothreitol and 10% trichloroacetic acid (for western blot studies), as described previously 43.

### Measurement of aortic geometry

Axial length, diameter and wall thickness were recorded prior to each experiment for calculation of vessel cross‐sectional area (CSA), which was subsequently used to measure vessel stress. Axial length and diameter were measured under a light microscope (×4) after aortic dissection of unloaded segments in TCB. For wall thickness measurements, ~1 mm length rings were cut at the proximal and distal end of each aortic ring and incubated in nuclear stain (NucBlue; Life Technologies, Carlsbad, CA, USA) for 30 min. After incubation, individual rings were imaged with fluorescence microscopy (×20) for autofluorescence of medial elastin fibres and NucBlue stain *via* NIS‐Elements software (Nikon Instruments, Melville, NY, USA). Approximately 15–20 measurements were recorded for each ring to calculate the average wall thickness. Subsequently, the averages for the proximal and distal rings for each strip were calculated to obtain the wall thickness for the upper and lower thoracic aorta.

### Cell culture

A7r5 rat aortic smooth muscle cells (ATCC, Manassas, VA, USA) were cultured in DMEM high glucose with 10% foetal calf serum, 1% glutamine, 50 units/ml penicillin and 50 μg/ml streptomycin. When A7r5 cells are serum‐starved, they express many smooth muscle‐specific markers, such as α‐actin, smooth muscle myosin, smooth muscle tropomyosin isoforms, h1 calponin and SM22α 44, 45. We favour this approach to the use of primary cultured cells due to the variability of differentiation states with passages. Freshly dissociated cells were not an option for these experiments since they only live 6–9 hrs after isolation. Cells were grown to confluency and then serum‐starved for 24 hrs prior to experimentation.

### Transfection of cells

**Figure 2:**
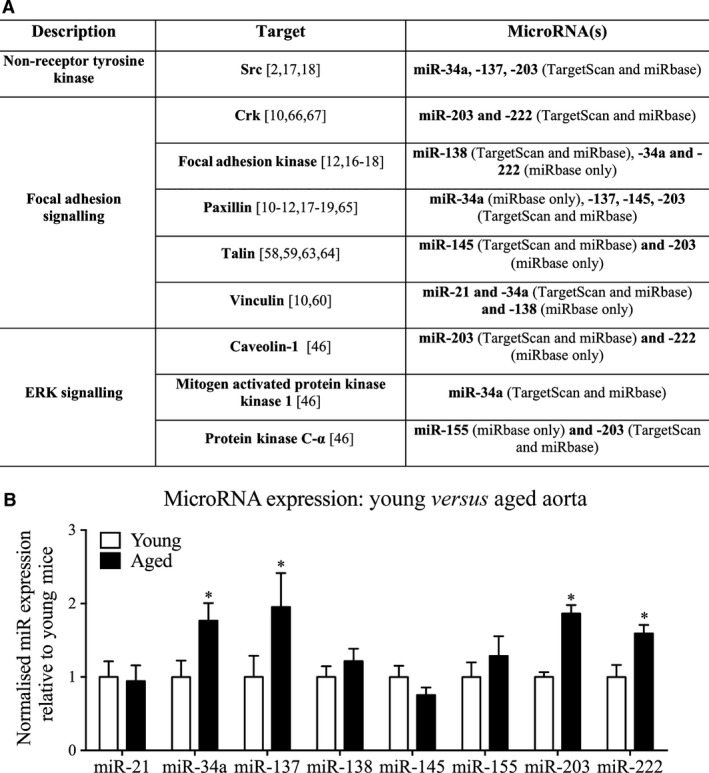
Ageing alters the expression of actin cytoskeletal‐ and focal adhesion‐regulating microRNAs in the mouse thoracic aorta. (**A**) TargetScan and miRbase were used to identify miRs that could potentially regulate expression of genes involved in FA and ERK signalling pathways, by reverse target prediction (*i.e*. using the gene targets to select the miRs of interest). (**B**) The expression level of miRs identified in the above table were compared from young (3 months) and aged (24–29 months) mice (*n* = 12 mice). *P < 0.05 (two‐tailed Student's *t*‐test).

A7r5 cells were seeded at a density of 2 × 10^5^ in 6‐well pates. Cells were transfected using 0.2% Lipofectamine RNAiMAX 3000 reagent, mixed with hsa‐miR‐203 miRvana miR mimic or scrambled miR mimic negative control for 24 (RNA quantification) or 72 hrs (protein quantification). Both hsa‐miR‐203 mimic and scrambled control were diluted in Opti‐Mem medium (Gibco, ThermoScientific, Cambridge, MA, USA) at a final concentration of 5 nM. For results presented in Figure 2, cells were stimulated with 12‐deoxyphorbol 13‐isobutylate 20‐acetate (DPBA) for 10 min. (as described previously Ref. 46). Since DPBA was diluted in dimethylsulphoxide (DMSO), which may influence ERK1/2 phosphorylation 47, 48, 49, equimolar DMSO (0.03%) was added to unstimulated cells as a vehicle control.

### Transfection of aortic tissue

Aortas were harvested from 3‐month‐old mice, cut into 2–3 mm pieces and placed in 24 well plates with 1 ml Opti‐Mem medium. Rings were transfected overnight in Opti‐Mem supplemented with 0.2% Lipofectamine RNAiMAX 3000 reagent and hsa‐miR‐203 miRvana miR mimic or scrambled miR mimic negative control (100 nM). The following day, rings were transferred into new 24 well plates containing a 1:1 mixture of TCB and Opti‐Mem in the presence of penicillin (25 U/ml) and streptomycin (25 mg/ml) and the medium was changed every day. On the fourth day of serum‐free organ culture, rings were prepared for measurement of *ex vivo* aortic stiffness (see below).

### *Ex vivo* aortic stiffness measurement with high‐frequency, low‐amplitude stretches

Aortic rings were mounted with two triangular pieces of wire (0.01 inch diameter). The lower triangle was attached to a micrometer (allowing adjustment of stretch during normalization) and the upper triangle was attached to a force transducer (which recorded changes in vessel wall tension). Once mounted, the vessels were incubated in organ baths containing oxygenated (95% O_2_ – 5% CO_2_) physiological salt solution (PSS; Krebs solution – in mM: 120 NaCl, 5.9 KCl, 1.2 NaH_2_PO_4_, 25 NaHCO_3_, 11.5 dextrose, 1 CaCl_2_, and 1.4 MgCl_2_; pH = 7.4). Stretch was monitored by the model 300C Dual‐ Mode Lever Arm System by Aurora Scientific (Aurora, ON, Canada) and performed using an electronic input to the lever arm motor *via* a function generator. Chart software (AD Instruments, Sydney, Australia) was used for data acquisition from the system. Each ring was stretched to optimal length L_O_ (1.8× slack length) 2 and left to equilibrate for 30 min. Vascular smooth muscle viability was confirmed by depolarization for 15 min. through the addition of PSS, in which 51 mM NaCl had been replaced by KCl, followed by return to PSS for 30 min. Since we were interested in activated smooth muscle cell stiffness, measurements at L_O_ were collected at baseline and 15 min. after stimulation with the α‐adrenoreceptor agonist phenylephrine (PE; 10 μM).

*Ex vivo* aortic stiffness is defined in this study as the material stiffness of aortic tissue, equivalent to the elastic modulus calculated from circumferential force responses to small‐length oscillations (as described previously Refs 2, 18, 50, 51). This method has previously been determined to result in a negligible phase lag of the force response, and does not break actomyosin crossbridges in the aortic tissue 50. Briefly, high frequency (40 Hz), low amplitude (+1% strain from L_O_) sinusoidal stretching was applied to produce a force response from which the change in stress could be measured as the amplitude of force normalized to CSA. Stiffness (E, kPa), can then be calculated as the change in stress divided by the change in strain (+1% strain from L_O_): E = (ΔF/A)/(ΔL/L_0_), where ΔF is the amplitude of the force response to the cyclic stretches, A is the CSA, ΔL is the amplitude of the cyclic stretches, and L_0_ is the optimal length.

### 5‐aza‐2′‐deoxycytidine assay

To test if promoter demethylation can cause miR‐203 overexpression, we treated cells with a hypomethylating agent (as described previously Refs. 52, 53). A7r5 cells were seeded into 6‐well plates at a density of 2 × 10^5^ and treated with 1.5 μM of the DNA methyltransferase (DNMT) inhibitor 5‐aza‐2′‐deoxycytidine (5‐Aza) or vehicle control (DMSO) for 72 hrs. Cells were then harvested for quantitative RT‐PCR analysis of miR‐203 expression (see below).

### RNA quantification

RNA was isolated with a Trizol‐based protocol at 4°C. For tissue, quick‐frozen aortic strips were homogenized three times at 2500 g for 15 sec. by a beat‐beating technology (Precellys 24; Bertin Technologies, Montigny‐le‐Bretonneux, France). For cell culture, cells were scraped from the cell culture plates directly in Trizol and then mixed several times. RNA was quantified by NanoDrop (Agilent Technologies, Santa Clara, CA, USA) and a 260/280 ratio of >1.8 was required for further study. RNA was reverse transcribed by the TaqMan MicroRNA Reverse Transcription or TaqMan Reverse Transcription kits for the quantification of miR and gene expression, respectively, according to the manufacturer's instructions. TaqMan miR assay kits were used for miR‐21 (5′‐UAGCUUAUCAGACUGAUGUUGA‐3′), miR‐34a (5′‐UGGCAGUGUCUUAGCUGGUUGU‐3′), miR‐137 (5′‐UUAUUGCUUAAGAAUACGCGUAG‐3′), miR‐138 (5′‐AGCUGGUGUUGUGAAUCAGGCCG‐3′), miR‐145 (5′‐GUCCAGUUUUCCCAGGAAUCCCU‐3′), miR‐155 (5′‐UUAAUGCUAAUUGUGAUAGGGGU‐3′), miR‐203 (5′‐GUGAAAUGUUUAGGACCACUAG‐3′), miR‐222 (5′‐AGCUACAUCUGGCUACUGGGU‐3′) and RNU6 (control). For mRNA analysis, TaqMan mouse gene expression assays were used for caveolin‐1 (Mm00483057_m1), Crk (Mm00467065_m1), Dnmt1 (Mm01151063_m1), Dnmt3a (Mm00432881_m1), Dnmt3b (Mm01240113_m1), paxillin (Mm00448533_m1), protein kinase C‐α (Mm00440858_m1), Src (Mm00436785_m1) and talin‐2 (Tln2; Mm00659397_m1). Gene expression was normalized to GAPDH (for tissue) or 18S (for cell culture). Quantitative reverse transcription polymerase chain reaction (qRT‐PCR) was performed on the StepOnePlus Real‐Time PCR system (Applied Biosystems, Waltham, MA, USA). All fold changes were calculated by the ΔΔCt method as described previously 54 and compared with either young mice (in ageing comparisons), scrambled control (in miR mimic comparisons) or vehicle control (in 5‐Aza comparisons).

### Western blotting

**Figure 3:**
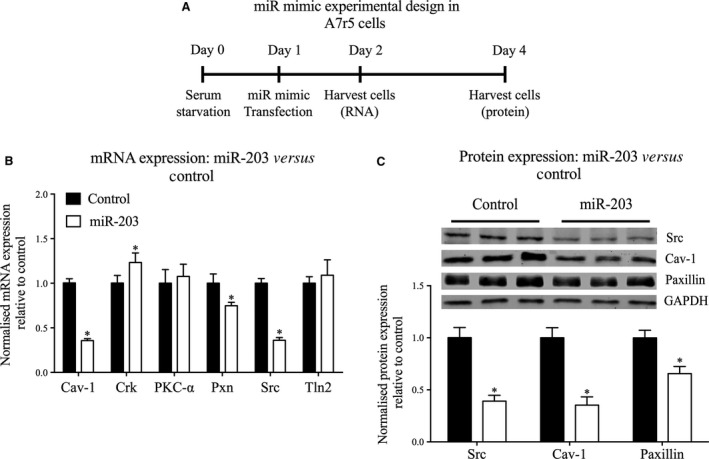
Overexpression of miR‐203 decreases both mRNA and protein levels of predicted targets in A7r5 cells. (**A**) Schematic of the protocol for transfection of A7r5 cells with miR‐203 mimic or control mimic. (**B**) mRNA expression levels for Cav‐1, Crk, PKC‐α, Pxn, Src and Tln2 compared in cells transfected with miR‐203 mimic or control (*n* = 6 experiments). (**C**) (Top) Representative western blots depicting Src, Cav‐1 and paxillin expression in control and miR‐203 mimic transfected cells. (Bottom) Normalized expression of Src, Cav‐1, and paxillin (*n* = 12 experiments). *P < 0.05 (two‐tailed Student's *t*‐test).

**Figure 4:**
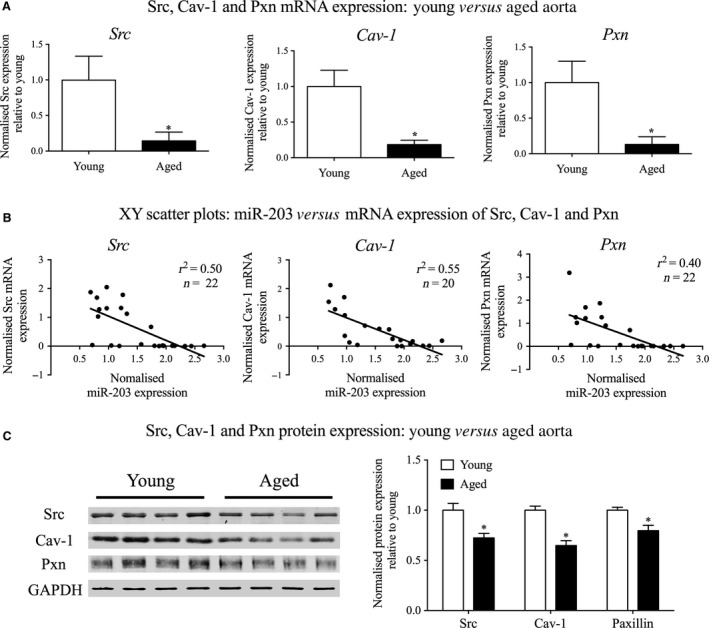
Ageing regulates the expression of miR‐203 targets in mouse aorta. mRNA and protein expression of the validated miR‐203 targets were analysed to determine if ageing regulated their expression in the thoracic aorta. (**A**) mRNA expression levels of Src, Cav‐1 and Pxn compared in the thoracic aorta from young and aged mice (*n* = 10–11 mice). (**B**) XY scatter plots generated to compare the levels of miR‐203 with the mRNA levels of its targets in the aorta from each individual mouse. (**C**) Typical western blots depicting Src, Cav‐1 and paxillin protein expression in young and aged aorta are shown on the left, with the subsequent quantification comparing their normalized expressions on the right (*n* = 8 mice). *P < 0.05 (two‐tailed Student's *t*‐test).

**Figure 5:**
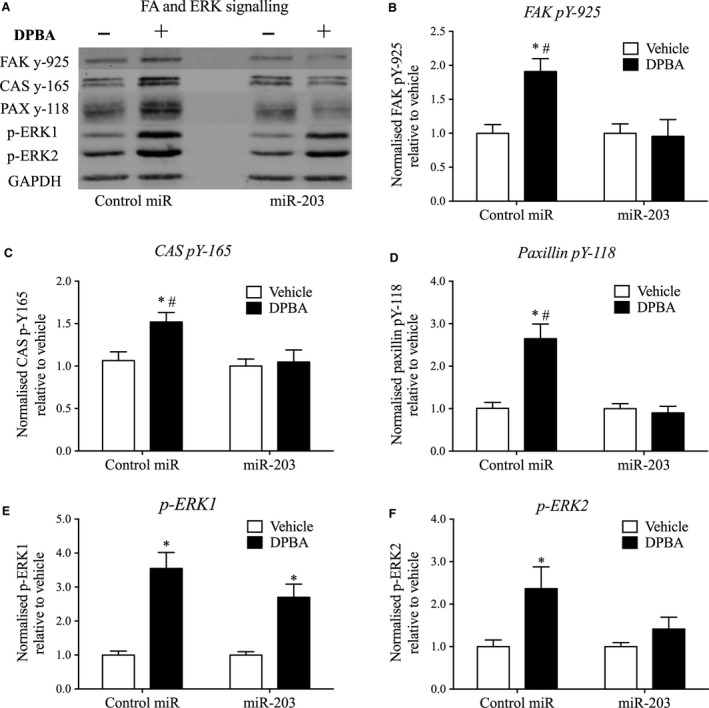
miR‐203 overexpression downregulates agonist‐induced FA and ERK signalling. A7r5 cells were stimulated with DPBA (3 μM for 10 min.) 72 hrs post‐transfection to determine the influence of miR‐203 on signalling pathways downstream of Src. (**A**) Typical western blots depicting FA signalling in cells transfected with miR‐203 or control mimic. DPBA‐induced phosphorylation of FAK pY‐925 (**B**), CAS pY‐165 (**C**), paxillin pY‐118 (**D**), p‐ERK1 (**E**) and p‐ERK2 (**F**) compared in A7r5 cells (*n* = 7 experiments) transfected with miR‐203 or control mimic. *P* < 0.05 compared to vehicle control (*) and negative miR mimic control (#) (two‐way anova).

Mouse aortic tissue was homogenized in a buffer containing 20 mM MOPS, 4% SDS, 10% glycerol, 10 mM DTT, 20 mM β‐glycerophosphate, 5.5 μM leupeptin, 100 μM pepstatin, 20 KIU aprotinin, 2 mM Na_3_VO_4_, 1 mM NaF, 100 μM ZnCl_2_, 20 μM AEBSF, and 5 mM EGTA. Quick‐frozen aortic strips were homogenized ten times at 1500 g for 25 sec. (see above section). Samples were placed on ice between cycles. Cells were harvested on ice for 30 min. by cell‐scraping directly in lysis buffer (mmol/l: 140 NaCl, 3 MgCl_2_, 1 dithiothreitol and 0.5% Nonidet‐P40 in a 20 mmol/l sodium phosphate buffer, pH 8.0) supplemented with protease inhibitor cocktail (Roche, Indianapolis, IN, USA), and mixed several times. Cell lysates were cleared by centrifugation (16,100 g 10 min. at 4°C). Fifteen μg of protein was resolved by SDS‐PAGE, transferred to a nitrocellulose membrane for western blotting with the following antibodies: Caveolin‐1, paxillin, Src, CAS p130, p‐ERK1/2, p‐FAK Tyr925, p‐Paxillin Tyr118 or GAPDH and viewed following incubation with IRDye 680 or IRDye 800CW labelled goat anti‐rabbit or goat‐anti‐mouse IgGs. Protein bands were visualized on an Odyssey infrared imaging system (LI‐COR, Lincoln, NE, USA) and densitometric analysis was performed with the Odyssey 2.1 software (LI‐COR, Lincoln, NE, USA). Intensity was adjusted for display purposes but all analysis was performed on the raw data. For analysis of all protein expression, bands of interest were normalized to GAPDH. Bands were further normalized to young aorta (Fig. 4), scrambled miR mimic control (Fig. 3) or unstimulated cells (Fig. 5).

### Reagents and antibodies

General laboratory reagents were obtained at analytic grade from Sigma‐Aldrich (St. Louis, MO, USA) and Bio‐Rad (Hercules, CA, USA). The α‐adrenoreceptor agonist PE and hypomethylating agent 5‐Aza were purchased from Sigma‐Aldrich. Cell culture reagents were acquired from Invitrogen and Gibco (ThermoScientific, Cambridge, MA, USA). For the stimulation of A7r5 cells, DPBA (LC Laboratories, Woburn, MA, USA) was used at a final concentration of 3 μM. The following antibodies were used: caveolin‐1 (Cav‐1; #D46G3 rabbit monoclonal 1:2000), p‐ERK1/2 (#T202/Y204 rabbit 1:1000), p‐FAK Tyr925 (#Y925 rabbit 1:200), p‐Paxillin Tyr118 (#Y118 rabbit 1:200) (from Cell Signaling, Danvers, MA, USA), paxillin (#610052 mouse monoclonal 1:1000), CAS p130 (#610271 mouse 1:250) (from BD Transductions, San Jose, CA, USA), Src (#sc‐18 rabbit 1:500; Santa Cruz Biotechnology, Santa Cruz, CA, USA; for aortic tissue) or (#AM77189 mouse monoclonal 1:500; Abgent, San Diego, CA, USA; for A7r5 cells) and GAPDH (#G9545 rabbit 1:20,000; Sigma‐Aldrich). IRDye labelled secondary antibodies were purchased from LI‐COR (1:1000). RNA‐based experimental reagents, including Trizol, Taqman and miR mimic kits were acquired from Life Technologies, ThermoScientific.

### Statistics

All values are presented as mean ± S.E.M. Analysis was carried out using the GraphPad Prism (7.0) software (La Jolla, CA, USA). For biochemical analysis (of mRNA and protein expression) and stiffness comparisons, groups were compared using a Student's *t*‐test (two‐tailed) for parametric data. DPBA‐induced phosphorylation was compared using a two‐way anova. For grouped analyses, data were analysed with Sidak's multiple comparisons test. Significance was assumed at *P* < 0.05.

## Results

### *In vivo* aortic stiffness increases with age in the mouse

Pulse wave velocity is the gold standard for clinical assessment of aortic stiffness. Aortic stiffness is well‐known to increase with age in humans, beginning around age 50 55. However, to the best of our knowledge only a single paper has appeared demonstrating an age‐induced increase in PWV in the mouse, and this measurement was at a single age point of 29 months 56. The age of 29 months in the mouse is reported to be comparable to ~80 years of age in the human 57. The question arises as to whether aortic stiffness in the mouse model has a similar time course to that of the human. Here, before beginning *in vitro* studies, we determined the ageing time course of the increases in *in vivo* aortic stiffness in the mouse. As shown in Figure 1B, PWV was similar in 4‐ and 10‐month‐old mice (similar to 20–40 years of age in humans) 57, but increased in 18‐ and 24‐month‐old mice (comparable to 56–69 human years) 57. Thus, similar to humans, increased aortic stiffness begins in middle age and continues into old age in the mouse. In the present studies we used mice between 24 and 29 months of age where ageing‐induced aortic stiffness is well‐established.

### Bioinformatic analysis identifies eight miRs that are high probability matches with the ageing phenotype

In order to determine whether aberrant expression of miRs could play a role in the previously described age‐related impairment of Src‐mediated mechanotransduction of vascular smooth muscle, we employed a reverse target prediction‐based bioinformatics method (Fig. 2A). First, we identified genes that encode multiple signalling molecules involved in the regulation of FAs and cytoskeletal plasticity in VSMCs from the literature 10, 11, 12, 16, 17, 18, 19, 46, 58, 59, 60, 61, 62, 63, 64, 65, 66, 67. Then we used two distinct bioinformatic tools, TargetScan and miRbase, to predict miRs with a high probability of regulating the expression of these targets. Figure 2A highlights these miRs, predicted to target genes encoding FA proteins that we have previously associated with the regulation of cytoskeletal plasticity in vascular smooth muscle. From this analysis, miR‐34a, ‐137, ‐138, ‐145, ‐155, ‐203 and ‐222 were predicted to target the master regulator, Src, the FA proteins FAK, paxillin, vinculin, talin and Crk and, the ERK signalling proteins, mitogen‐activated protein kinase 1, protein kinase C‐α (PKC‐α) and Cav‐1. Next, we tested these putative associations by qRT‐PCR (Fig. 2B).

### Ageing in the mouse aorta increases expression of four miRs that target members of the Src signalling network

The proximal aorta from young and aged mice was harvested for qRT‐PCR analysis and probed for the miRs predicted in Figure 2A. Ageing significantly increased the proximal aortic expression of miR‐34a, ‐137, ‐203 and ‐222 by 77%, 95%, 92% and 59%, respectively, whereas no significant differences were observed in the expression levels of miR‐21, ‐138, ‐145, and ‐155 (Fig. 2B).

### miR‐203 mimic regulates expression levels of Src, Cav‐1 and Paxillin in A7r5 cells

As indicated in Figure 2A, miR‐203 is predicted to target multiple proteins of interest, namely Cav‐1, Crk, PKC‐α, paxillin, Src and Tln2, whereas the other increased miRs are predicted to target only one or 2 of these proteins. We therefore chose to focus the current study on determining whether the age‐associated increase in miR‐203 may *cause* changes in smooth muscle FA signalling.

To modify miR‐203 expression, we transfected with a miR‐203 mimic, which mimics the sequence of mature endogenous miR‐203, to enhance its activity in A7r5 cells (protocol diagrammed in Fig. 3A). Smooth muscle cell lysates were harvested at 24 hrs post‐transfection for gene expression analysis by qRT‐PCR. The miR‐203 mimic induced 64%, 64% and 25% decreases in Src, Cav‐1 and paxillin mRNA levels, respectively, but had no significant effect on PKC‐α or Tln2 expression (Fig. 3B). Surprisingly, overexpression of miR‐203 significantly *increased* Crk gene expression by 23%. Protein levels of these targets were then analysed by immunoblots of lysates of smooth muscle cells transfected with the miR‐203 mimic for 72 hrs. The miR‐203 mimic significantly decreased protein levels of Src, Cav‐1 and paxillin by 61%, 65% and 35%, respectively (Fig. 3C).

These findings indicate that miR‐203 regulates the expression of important FA proteins previously associated with signalling and remodelling of the cytoskeleton of aortic smooth muscle.

### mRNA expression levels of miR‐203 targets are regulated by ageing in mouse aorta

We next returned to ageing studies and tested whether there is a correlation between ageing, endogenous miR‐203 and its validated targets (Fig. 4). Consistent with an increase in miR‐203 expression with ageing (Fig. 2), mRNA levels for all three miR‐203 targets are decreased in aged aorta (Fig. 4A). In addition, comparison of miR‐203 expression with the expression levels of its associated target mRNAs, from each individual sample, revealed a direct negative correlation between miR‐203 and Src, Cav‐1 and paxillin mRNA expression levels, albeit with high variability (*i.e*. low *r* ^2^ numbers) (Fig. 4B).

### Src, Cav‐1 and paxillin protein levels are significantly changed with ageing

Protein levels were quantitated by immunoblot. A typical blot is shown in Figure 4C, left‐hand panel with mean values plotted in the right‐hand panel. Src, Cav‐1 and paxillin protein expression were decreased in aged aorta by 28%, 35% and 20%, respectively. Of note is the fact that the percentage change in protein expression is quantitatively smaller than the change in mRNA expression with ageing. This is likely due to the well‐known increased stability of many proteins compared to mRNA 68.

Taken together, these results suggest that miR‐203, which increases with ageing, may potentiate age‐induced downregulation of Src, Cav‐1 and paxillin protein expression levels in the mouse aorta.

### miR‐203 impairs FA signalling in A7r5 cells in a cause‐and‐effect manner

Next, we examined the impact of an increase in miR‐203 expression on signalling events critical to FA dynamics and cytoskeletal plasticity 2, 17, 18, 69. To achieve this, we overexpressed miR‐203 in A7r5 cells for 72 hrs and quantitated phorbol ester (DPBA)‐induced tyrosine phosphorylation of the FA proteins CAS, FAK, and paxillin, and ERK activation (Fig. 5A–F). Overexpression of miR‐203 completely abolished agonist‐induced tyrosine phosphorylation of FAK pY‐925, CAS pY‐165, and paxillin pY‐118 in A7r5 cells (Fig. 5B–D). Furthermore, agonist‐induced ERK2 phosphorylation was also eliminated in miR‐203 transfected cells (Fig. 5F). However, ERK1 phosphorylation was not significantly affected by miR‐203 transfection in A7r5 cells (Fig. 5E).

Taken together, these findings suggest that increased miR‐203 expression is involved in the previously observed age‐related impairment of FA signalling, resulting in dysfunction of vascular smooth muscle cytoskeletal dynamics.

### miR‐203 increases *ex vivo* active smooth muscle stiffness in young moue aorta

**Figure 6:**
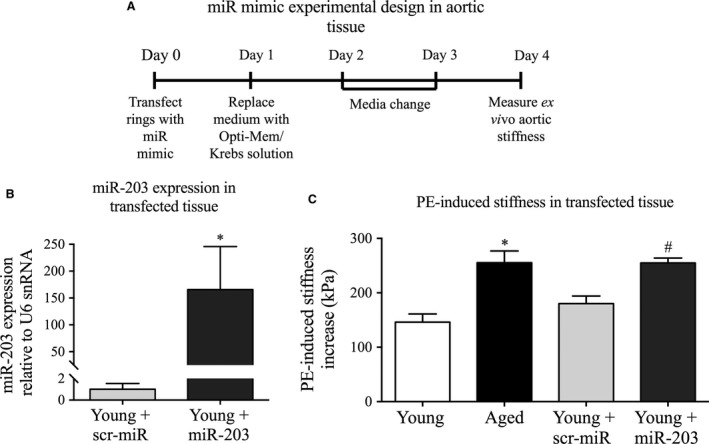
Overexpression of miR‐203 in young aorta reproduces the ageing phenotype of increased aortic stiffness. (**A**) Schematic of the protocol for transfection of aortic tissue with miR‐203 mimic or control mimic. (**B**) miR‐203 expression levels in tissue transfected with miR‐203 mimic or control (*n* = 4 experiments). (**C**) Agonist‐induced stiffness increase in young (white, *n* = 10) *versus* aged (black, *n* = 6) aorta and young aorta transfected with scrambled miR mimic control (light grey, *n* = 10) *versus* miR‐203 mimic (pattern grey, *n* = 9). *P < 0.05 from young aorta or # scrambled mimic control (two‐tailed Student's *t*‐test).

We next determined the effect of overexpressing miR‐203 on *ex vivo* stiffness of young mouse aorta. Similar to the method for transfecting smooth muscle cells, we transfected excised aortic rings for 72 hrs and then assessed PE‐induced aortic stiffness increase. We have previously experienced difficulty in transfecting vascular tissue from larger animals. However, as shown in Figure 6B, miR‐203 was significantly overexpressed in mouse aortic rings transfected with the miR‐203 mimic compared to scrambled miR mimic control. Interestingly, miR‐203 overexpression enhanced PE‐induced stiffness increases in young aorta (stiffness increase for control: 182.0 ± 19.4 kPa and miR‐203: 262.3 ± 12.0 kPa) to a similar extent as observed with ageing (stiffness increase for young: 151.1 ± 16.6 kPa and aged: 249.0 ± 27.6 kPa) (Fig. 6C).

These findings are consistent with increased miR‐203 expression in aged aorta playing a role in the age‐related increase in active smooth muscle stiffness.

### Ageing negatively regulates mediators of DNA methylation

**Figure 7:**
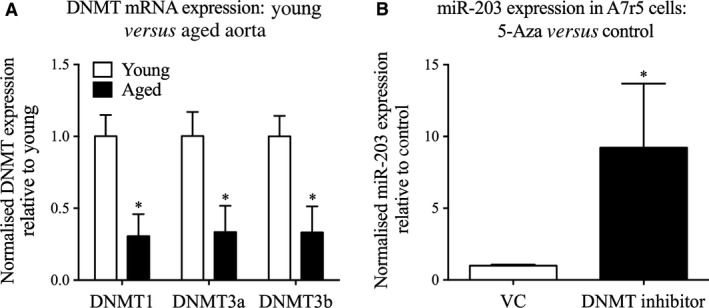
DNA methyltransferases (DNMTs) influence miR‐203 expression. (**A**) Gene expression of DNMT1, 3a and 3b in young (white) and aged (black) proximal mouse aorta (*n* = 10 mice). *Significant difference from young mice (two‐tailed Student's *t*‐test). (**B**) miR‐203 expression in cells treated with the DNMT inhibitor 5‐Aza for 72 hrs and harvested for quantification of miR‐203 expression (*n* = 6 experiments). *P < 0.05 (two‐tailed Student's *t*‐test).

The question arises as to the molecular mechanism by which miR‐203 levels are regulated by ageing. Previous studies, in other systems, have shown that the expression of miR‐34a, ‐137 and ‐203 are regulated by epigenetic methylation events in their respective promoter regions 82. We therefore hypothesized that aberrant expression of DNMTs played a role in the age‐related regulation of miR‐203 expression in vascular smooth muscle. As illustrated in Figure 7A, the mRNA levels of the three main DNMTs, DNMT1, 3a, and 3b, are significantly decreased in the aorta from aged mice by 68%, 65% and 67%, respectively. In addition, treatment of A7r5 cells with the DNMT inhibitor, 5‐Aza, increased miR‐203 expression several‐fold (Fig. 7B). These findings support the premise that age‐related hypomethylation of the miR‐203 promoter may cause the observed increase in its expression in aged aorta.

## Discussion

**Figure 8:**
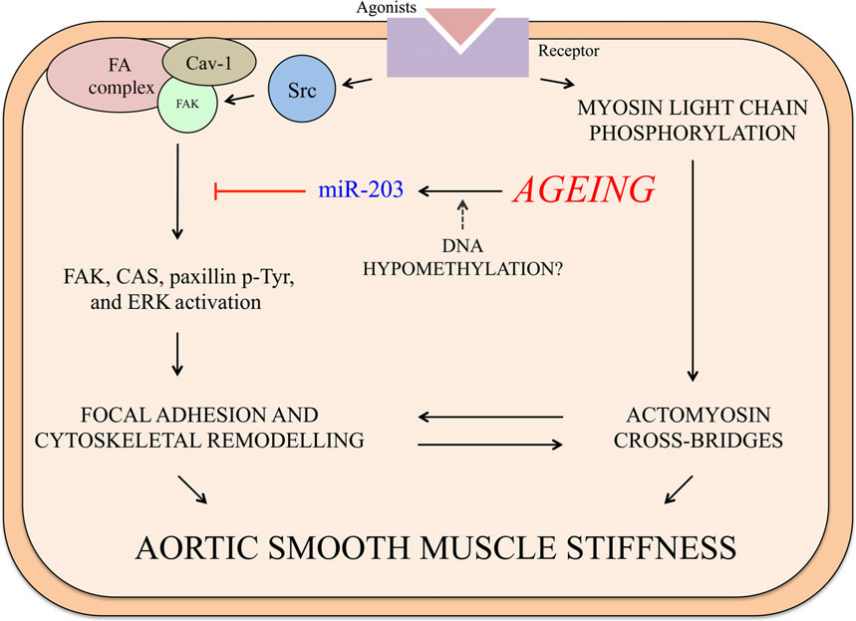
Proposed mechanism. Ageing triggers an increase in miR‐203 expression, leading to a reduction in Src‐mediated signalling. The age‐related increase in miR‐203 expression, at least partially caused by hypomethylation of the miR‐203 promoter region, negatively influences the expression of Src, Cav‐1 and paxillin. Consequently; downstream tyrosine phosphorylation (p‐Tyr) of FAK, paxillin and CAS and ERK activation are impaired, contributing to the age‐related defect in FA remodelling and impaired regulation of stiffness.

In this study we demonstrate, for the first time, that age‐related increases in miR‐203 expression in mouse aorta are associated with downregulation of critical FA signalling proteins in the VSMC, providing a mechanistic basis (summarized in Fig. 8) for the previously described defect in FA signalling with aortic ageing 2. In addition, *in vivo* data confirm that ageing is also associated with increased aortic stiffness in this mouse model, as measured by the gold standard for clinical assessment of stiffness, PWV. Interestingly, ageing and miR‐203 overexpression in young aorta similarly increased *ex vivo* measurements of activated smooth muscle stiffness. It appears, therefore, that miR‐203 contributes to the impairment of FA signalling in aged aorta, which is thought to be critical in the normal functioning of the proximal aorta as a buffer against the high systolic pressures generated by the heart.

In this study, we found that miR‐203 expression is increased in aged aorta, and since it is predicted to target many interesting FA‐related proteins of interest, we further examined its role in aortic smooth muscle cells. We also demonstrated that, consistent with the observed increase in miR‐203 expression, ageing was associated with a decrease in expression of its confirmed targets Src, Cav‐1 and paxillin at both the message and protein levels. These proteins are known to play an essential role in vascular smooth muscle function 10, 17, 19, 70, 71, 72, 73, 74, 75. Unexpectedly, Crk message levels increase following miR‐203 transfection. This is an intriguing finding, since miRs ordinarily negatively regulate target expression. Notwithstanding the possibility that miR‐203 downregulates an unknown repressor of Crk expression, miRs can also directly increase gene expression by interacting with promoter sequences 76. Although this is thought to be a rare occurrence, the influence of miR‐203 on Crk expression warrants further investigation.

It has recently been shown that the expression of miR‐203 is under the influence of methylation events in diverse animals and tissues 52, 53, 77, 78, 79, 80, 81, 82, 83. It is therefore reasonable to speculate that an age‐induced alteration in DNA methylation of the miR‐203 promoter might contribute to the increase in its expression. Indeed, we show here that the expression of key regulators of DNA promoter methylation, DNMTs, are decreased with ageing. Furthermore, we show that cells treated with a DNMT inhibitor display increased expression of miR‐203. Hence, hypomethylation, which is known to occur with ageing 84, could be the cause of this aberrant miR expression in aged aorta. Future experiments will be needed, however, to confirm this observation by specifically examining miR‐203 promoter methylation.

We report here that miR‐203 plays a role in the regulation of VSMC stiffness with increased ageing. Aortic stiffness, as measured *in vivo* by PWV, was increased at or before 18 months of age, which is consistent with the well‐established observation of increased aortic stiffness in humans between 50 and 60 years of age. Changes in aortic stiffness have often assumed to be dominated by alterations in the ECM. However, we and others have found that the activated smooth muscle contributes to overall aortic stiffness, challenging this dogma 2, 22, 23, 24, 25, 26, 27. Further, we find here that ageing increases activated (PE‐induced) smooth muscle stiffness of aortic rings, supporting the notion that changes at the level of the VSMC could lead to increased aortic stiffness. Of particular importance was the further finding that miR‐203 overexpression in young aorta also increased active aortic stiffness, therefore mimicking the aged phenotype. It should be noted that the approach used in the current study, miR mimic transfection, exaggerates the overexpression of miR‐203, although much of the transfected miR may not actually become incorporated into the RNA‐induced silencing complex 85. While it would be beneficial to study the chronic effects of smaller increases in miR‐203, it is not possible to keep tissue alive *ex vivo* for the required length of time. Further work will be required to determine the long‐term consequence of miR‐203 overexpression through genetic approaches 86. Despite this caveat, the data presented here point to the increase in miR‐203 expression playing a significant role in increased aortic stiffness with ageing.

The finding that an impairment of Src‐mediated signalling is associated with increased aortic stiffness is interesting. In contrast with our finding, FAK/Src‐mediated signalling has been found to directly increase ECM stiffness in fibroblasts 87, 88, and changes in FAK and CAS protein levels were found to increase cell stiffness 88, 89. However, it is important to point out that in the current study we did not actually see a change in FAK/CAS protein levels, but rather a change in Src‐dependent FAK/CAS signalling. Thus, for the ageing smooth muscle cell we propose an alternative mechanism. Our data are consistent with a model where an inactive FA signalling pathway, primarily caused by age‐ and miR‐203‐related reduction in Src expression, leads to impaired plasticity of VSMCs (Fig. 8). We have previously demonstrated that Src tyrosine kinase is a key player in the regulation of VSMC stiffness, through its effects on a program of FA and actin cytoskeletal remodelling pathways. The tyrosine phosphorylation of the FA proteins CAS pY‐165, FAK pY‐925, and paxillin pY‐118 causes their redistribution, which we previously found to occur through an endocytic recycling pathway 28, allowing cyclic remodelling of the FAs 10, 17, 69, 71. Interestingly, knockout of the Src gene supresses tyrosine phosphorylation, which is associated with their defective turnover and cyclic remodelling 90. We suggest that this cyclic remodelling process, which is lost with ageing, is critical for the proximal aorta to function as a shock absorber against the pulsatile forces from the heart 2.

The regulation of VSMC stiffness has also recently been shown to involve the ERK activation pathway 24. We show here that miR‐203 overexpression disrupts the activation of ERK2 in aortic smooth muscle cells, supporting a recent finding in airway smooth muscle 91. In this study, miR‐203 was found to downregulate the expression of Abl, a Src‐dependent kinase, which modulates ERK activation and, in turn, inhibits cell proliferation 91. In contractile smooth muscle, however, ERK activation promotes phosphorylation and inactivation of the actin‐binding protein caldesmon, alleviating caldesmon‐induced inhibition of myosin‐actin interactions, thus promoting VSMC contraction 92, 93, 94. Furthermore, a pathway linking Src‐dependent FA signalling with an ERK scaffolding function of FAs has recently been implicated in the regulation of VSMC stiffness 24. The molecular mechanism leading to miR‐203‐induced impairment of ERK activation are likely complex, but could be caused by the downregulation of the ERK scaffold Cav‐1, which facilitates the assembly of signalling cascades 46, 72. Further to its role as an ERK scaffold, Cav‐1 has recently been suggested to directly interact with Src to mediate FA signalling 95, 96. In this scenario, Src is scaffolded to lipid rafts by directly binding to Cav‐1, which leads to Cav‐1 phosphorylation at Y14. FA signalling promotes FAK autophosphorylation, which induces Src/Cav‐1 to relocalize to FAK‐containing FA complexes. Subsequent phosphorylation of FAK at Y925, which was abolished by miR‐203 in the present study, and Cav‐1 dephosphorylation are then important events in FA turnover and cell motility 95, 96. In a separate study, the interaction between Cav‐1 and Src was found to be essential for the accumulation of Src in FAs 97. It was interesting, therefore, that Cav‐1 protein was downregulated in the current study, which likely contributes to the defective FA signalling we have reported previously 2. When taken together, these results suggest that miR‐203 regulates the expression of Src, Cav‐1 and paxillin, thereby disrupting the dynamic nature of FA remodelling pathways, which leads to dysregulation of VSMC stiffness in aged aorta (Fig. 8). Focal adhesion signalling is highly complex, however, and it is likely that changes to both inside‐out and outside‐in signalling, involving critical relationships between the ECM, integrins, and the actin cytoskeleton, are responsible for changes in VSMC stiffness. Furthermore, the development of aortic stiffness is clearly multifaceted and involves changes in ECM composition as well as to the intrinsic properties of endothelial and smooth muscle cells.

Although we have focussed our detailed analysis on miR‐203 in the present study, we did find that miR‐34a, ‐137 and ‐222 were also significantly upregulated in aged mouse aorta. Thus, ageing could alter an entire program of VSMC signalling. This also supports previous studies in aged mouse aorta, which have reported an association between miRs that trigger increased inflammatory signalling 98, 99. We confirmed the previously reported increase in miR‐34a expression in aged mouse aorta, which is associated with decreases in the well‐studied longevity gene Sirtuin 1 98. Interestingly, miR‐34a is also known to target vinculin 100, which links the transmembrane integrins to the actin filaments *via* talin interactions, and could therefore also be an important player in the regulation of VSMC stiffness and contractility 10, 58, 63. This is the first study to find an association between vascular ageing and miR‐137 expression. It is important to note that both miR‐34a and ‐137 are also predicted to target Src and paxillin. Furthermore, several studies have suggested a role for miR‐222 in VSMC function 101, 102, 103, 104, including an association with vascular smooth muscle contractility changes in diabetic rats 102. Further studies will be required to address the functional implications in multiple disease‐associated settings of the age‐related increase in miR‐34a, ‐137 and ‐222 expressions in aortic smooth muscle.

In summary, we show here that male C57BL/6 mice develop aortic stiffness at or before 18 months of age, which is consistent with the well‐established observation of increased aortic stiffness in humans between 50 and 60 years of age. Ageing increases the expression of miR‐203, possibly through promoter hypomethylation, which leads to a decrease in Src, Cav‐1 and paxillin expression. Overexpression of miR‐203 impairs agonist‐induced FA signalling in aortic smooth muscle cells, and increases VSMC stiffness of aortic tissue from young mice. We have previously shown that the impairment of Src‐mediated FA signalling disrupts a VSMC stiffness regulatory pathway in aortas from aged mice 2. Hence, miR‐203 is an upstream mediator of this defective regulation, which interferes with the ability of the aorta to modulate pulsatile pressures coming out of the heart. In conclusion, this study provides novel insights into the molecular mechanisms of age‐related impairment of VSMC function and points to potential therapeutic targets for the modification of aortic stiffness development with ageing.

## Conflict of interest

The authors confirm that there are no conflicts of interest.

## Author contributions

C.J.N. conceived, designed and performed the experiments, analysed and interpreted the data, and co‐wrote the manuscript. F.S. and S.L. designed and performed the experiments and helped interpret the results. K.G.M. conceived and designed the experiments and interpreted the data, and co‐wrote the manuscript. All authors approved the final version of the manuscript.
